# A risk-benefit assessment of dietary selenium and its implications in preschool children’s growth performance in Taiwan

**DOI:** 10.1265/ehpm.25-00128

**Published:** 2026-01-09

**Authors:** Chi-Sian Kao, Ying-Lin Wang, Chuen-Bin Jiang, Ying-Chih Chuang, Yi-Hua Chen, Hsing-Jasmine Chao, Pin-Hsuan Lin, Hsing-Cheng Hsi, Ling-Chu Chien

**Affiliations:** 1 School of Public Health, Taipei Medical University, Taipei, Taiwan; 2 Graduate Institute of Environmental Engineering, National Taiwan University, Taipei, Taiwan; 3 Department of Pediatric Gastroenterology, Hepatology and Nutrition, MacKay Children’s Hospital, Taipei, Taiwan; 4 Department of Medicine, College of Medicine, MacKay Medical University, New Taipei City, Taiwan; 5 Neuroscience Research Center, Taipei Medical University, Taipei, Taiwan; 6 Nutrition Research Center, Taipei Medical University Hospital, Taipei, Taiwan

**Keywords:** Selenium, Mercury, Food, Risk-benefit assessment, Nutrient

## Abstract

**Background:**

Optimizing nutrient intake is crucial for the health and development of preschool children. While previous studies assessed the risks and benefits of selenium (Se) from fish and seafood, few have examined its intake from common foods and its association with children’s growth.

**Objective:**

In this study, we evaluated optimal foods for achieving a dietary Se surplus and the implications for the growth performance of preschool children.

**Methods:**

Mercury (Hg) and Se concentrations were analyzed in 108 commercially available foods to conduct a dietary risk-benefit assessment of Se intake. Hg exposure was evaluated using hair samples from 349 preschool children enrolled between August 2017 and July 2022. Information on food consumption frequencies and nutrient compositions was obtained through dietary surveys.

**Results:**

Overall, 42.4% of children had hair Hg levels above the US Environmental Protection Agency’s reference dose of 1 µg/g, showing that Hg exposure among preschool children in Taiwan remains a significant issue. The risk-benefit assessment revealed that eggs and fish are superior sources of Se compared to other animal- and plant-based foods. Although marine fish contained higher Hg concentrations than eggs, their relatively high Se and ω-3 polyunsaturated fatty acid (PUFA) contents supported a favorable nutritional profile when consumed in moderation. The minor negative health benefit value of Se (HBV_Se_) observed for fruit does not pose a health concern, as it is offset by other Se-rich foods in the diet. The cumulative HBV_Se_ across food groups indicated that the children’s overall dietary Se intake was positive and nutritionally advantageous. Dietary Se, mainly from fish and eggs, was positively associated with weight and height development, whereas excessive fruit consumption may slightly reduce Se intake and adversely affect growth.

**Conclusions:**

Moderate consumption of fish and eggs should be encouraged to support optimal growth and neurodevelopment. Overall, dietary patterns of Taiwanese preschool children provide beneficial levels of Se and ω-3 fatty acids while maintaining low Hg-related risks, emphasizing the need for continued monitoring of Hg levels in locally consumed foods to ensure dietary safety.

**Supplementary information:**

The online version contains supplementary material available at https://doi.org/10.1265/ehpm.25-00128.

## Graphical Abstract

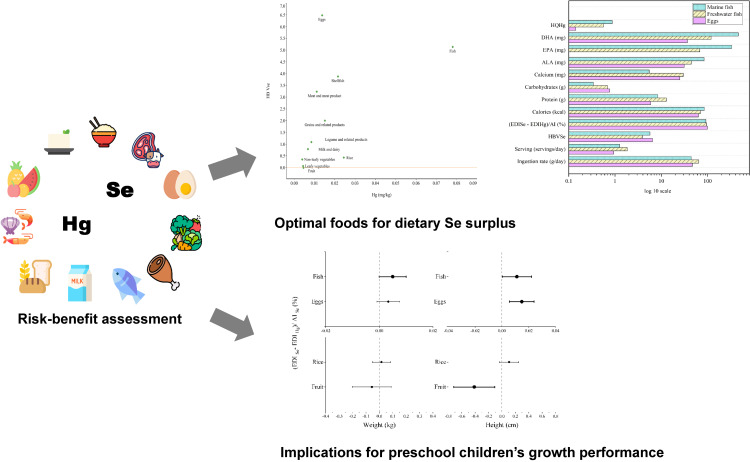

## 1. Introduction

Evaluating the health risks of dietary mercury (Hg) exposure is necessary, especially for vulnerable populations such as children and pregnant women [1–3]. Increasing attention has recently emphasized the importance of assessing the risk-benefit balance of dietary selenium (Se) intake, given its essential role in selenocysteine (Sec) synthesis and selenoenzyme activities that protect against oxidative damage. Excessive Hg exposure can bind to Se, forming inert Hg-Se complexes that render Se unavailable for further Sec synthesis, potentially leading to a functional Se deficiency and inhibition of selenoenzyme activity in brain and endocrine tissues [4, 5]. Maintaining adequate dietary Se intake is therefore critical to sustain selenoenzyme functions and mitigate the biochemical consequences of high Hg exposure.

Hg may exert adverse health effects, primarily due to its organic methylmercury (MeHg) form, which induces neurotoxic outcomes at high exposure levels [6, 7]. In aquatic and agricultural environments, inorganic Hg undergoes microbial methylation to form MeHg, accounting for approximately 90%–100% of total Hg accumulated in fish [8–10]. MeHg can easily penetrate the blood-brain barrier and placenta, posing significant risks to neurodevelopment, cognitive abilities, and growth in early life [11–13].

Few studies have investigated Hg exposure among preschool children. Our previous survey in Taiwan, which analyzed Hg concentrations in the hair of children under 6 years of age, highlighted significant concerns regarding early-life Hg exposure [14, 15]. Hair Hg concentrations are widely recognized as a reliable biomarker for assessing long-term exposure, as human hair grows at an average rate of approximately 1 cm/month [16]. Moreover, MeHg typically accounts for about 80% of total Hg in hair [17]. Therefore, hair serves as an important and robust indicator for evaluating Hg exposure in children.

Fish and seafood consumption is the primary medium of exposure to Hg for younger children [14, 18, 19], with previous results revealing that 80%–90% of Hg exposure comes from ingesting fish [20]. However, seafood serves as a valuable source of omega-3 polyunsaturated fatty acids (PUFAs), essential for children’s brain and neurodevelopment [21]. Coastal populations, such as those in the Mediterranean and Taiwan, are at increased risk for Hg exposure [14, 22]. Evaluating the risks and benefits of Hg and nutrition is critical for diet advisories and recommendations. Se is an essential trace element for human health [23], and is abundantly present in seafoods, meats, poultry, and eggs [24]. Adequate Se intake helps maintain selenoenzyme activity and counteracts the functional Se deficiency induced by Hg exposure [25]. Ralston et al. [26] proposed an improved equation for the health-benefit value of Se (HBV_Se_) index, which accounts for the impacts of both dietary Hg exposure and Se intake on Se levels. This index helps assess the overall advantage of Se consumption from food and the reduced adverse effects of Hg [27]. Many studies revealed a benefit-risk assessment of fish and seafood consumption [25, 28, 29]. However, few surveys evaluated the benefit-risk assessment of dietary foods and their implications for growth and development. Optimizing beneficial nutrient intake while decreasing exposure to Hg is crucial, especially for nutritionally vulnerable groups such as preschoolers and women of childbearing age.

Accordingly, the objectives of this study were to (1) evaluate total Hg (THg) and Se concentrations in foods commonly consumed by children; (2) investigate THg exposure through a hair analysis; (3) assess the risk-benefit of each food and whether it meets adequate daily Se intake; (4) evaluate the association between dietary Se surpluses and growth performance; and (5) identify optimal foods for nutritional health.

## 2. Materials and methods

### 2.1 Study population and food consumption data collection

In total, 349 preschool children were enrolled between August 2017 and July 2022. Data on participants’ demographic characteristics and food ingestion frequency were collected using a structured questionnaire. A food frequency questionnaire (FFQ) was used to collect detailed information about the types of food that children consumed over the past month. Parents were asked to report their child’s daily or weekly intake for each food category, as well as the serving size for each occasion, allowing for a thorough assessment of dietary habits. These data were then used to estimate the daily intake of THg and selenium (Se) and assess whether intake levels were adequate (Supplemental Fig. S1). Hair samples were collected from all children to assess the internal exposure dose of Hg. Taipei Mackay Memorial Hospital (18MMHIS028) approved this work. Before enrollment, the parents of each participant provided written informed consent.

### 2.2 Anthropometric survey data

Anthropometric indexes of weight (kg) and height (cm) were determined by well-trained field staff from medical centers when children were recruited. Z-scores, including the weight-for-age z-score (WAZ) and height-for-age z-score (HAZ), were calculated based on World Health Organization (WHO) Child Growth Standards. Therefore, we evaluated associations of benefit-risk assessments of Se with anthropometric outcome z-scores using regression models without adjusting for gestational age.

### 2.3 Hg and Se analyses

#### 2.3.1 Dietary food

Based on food categories reported in the FFQ, 108 food samples were collected and grouped into 11 categories. Analytical procedures were referenced from previous research conducted by Kao et al. [14]. THg levels were determined using cold-vapor atomic absorption spectrophotometry (CVAAS; HG-200, Hiranuma, Mito, Japan), while Se concentrations were measured with inductively coupled plasma-mass spectrometry (ICP-MS; Agilent 7800, Agilent Technologies, Santa Clara, CA, USA). Standard reference materials (SRMs), DORM-3 and GBW10012, were utilized to validate the analytical quality. The precision of THg and Se measurements respectively ranged 0.06%–16.8% and 1.1%–23.1%. The accuracy of these elements was 98.1%–107.6%. Method detection limits (MDLs), calculated as three times the standard deviation of the blank concentration, were 0.009 ng/g for THg and 0.03 ng/g for Se.

#### 2.3.2 Hair

THg contents in hair samples were measured following a modified US Environmental Protection Agency (EPA) method 1631E [30]. Hair samples were taken from the occipital region of the children, rinsed three times with deionized water, and dried at 37 °C. To each 0.1 g of hair sample was added 5 mL of 69% nitric acid (J.T. Baker^®^, Center Valley, PA, USA) in a water bath at 90 °C for 3 h. THg levels were analyzed using CVAAS (HG-200, Hiranuma). To ensure accuracy, the GBW09101b SRM was used, and precision was verified through analytical duplicates. The precision of THg measurements ranged 0.04%–6.8%, with accuracies of 92.1%–112%. The MDL for THg in hair was 0.013 ng/g.

### 2.4 Risk-benefit assessment

#### 2.4.1 Se:Hg molar ratio and Se’s health benefit value (HBV_Se_)

The Se:Hg molar ratio was calculated by the following equation:

$$\text{Se:Hg molar ratio}=\frac{\text{Se (µmol/kg)}}{\text{Hg (µmol/kg)}}.$$

The HBV_Se_ index more accurately demonstrates the net health benefit of dietary Se intake considering the hazard of dietary Hg. The index helps assess the surplus (positive values) or deficit (negative values) of Se levels associated with food ingestion and is calculated by the following equation [26]:

$${\text{HBV}}_{\text{Se}}=\frac{\left(\text{Se}-\text{Hg}\right)}{\text{Se}}\times (\text{Se}+\text{Hg});$$

where Se and Hg values applied in both equations are the molar concentrations (µmol/kg) of Se and Hg in food samples, respectively.

A Se:Hg molar ratio of >1 or an HBV_Se_ value of >0 indicates that Se may compensate for the toxicological risk from Hg exposure [31]. Because the HBV_Se_ equation considers the absolute and relative levels of Se and Hg, this revised equation helps account for disproportionately high values in food with extremely low Hg contents [26].

#### 2.4.2 Estimated daily intake (EDI) and adequate intake (AI) percentages

The EDI of dietary Hg was calculated by the following equation [32]:

$$\text{EDI}=\frac{C_{i}\times {\text{IR}}_{i}}{\text{BW}};$$

where C_i_ is the level of THg or Se (mg/kg) in each category, IR_i_ is the daily intake rate by children (g/day) based on the FFQ, i refers to each food category, and BW is body weight (kg) of a child. The EDI of Hg or Se through food consumption is expressed in µg/kg-BW/day.

According to the EDI, we calculated percentages of the Se AI (AI_Se_) recommended by the European Food Safety Authority (EFSA). The AI_Se_ for preschool children is 20 µg/day [33, 34].

#### 2.4.3 Health risk

The hazard quotient (HQ), calculated to evaluate potential non-carcinogenic risks associated with dietary Hg exposure [35], indicates a health risk if it exceeds a value of 1; such risks particularly impact children’s neuropsychological development [12, 36]. The HQ was calculated by the following equation:

$$\text{HQ}=\frac{\text{EDI}}{\text{RfD}};$$

where HQ is the hazard quotient, and the reference dose (RfD) for dietary Hg established by the Food and Agriculture Organization (FAO) and the World Health Organization (WHO) Joint Expert Committee on Food Additives (JECFA) is 0.23 µg/kg-BW/day [32].

The maximum safe daily dietary intake (CR_lim_) (g/day) was estimated following guidelines of the US EPA [37] and was calculated using the following equation:

$${\text{CR}}_{\text{lim}}=\frac{\text{RfD}\times \text{BW}}{\text{Ci}};$$

where RfD is the reference dose of Hg or Se (Hg: 0.0001 mg/kg/day; Se: 0.005 mg/kg/day), C_i_ is the concentration of THg or Se (mg/kg) in each food category, and BW is the body weight (kg) of a child (13.87 kg in this study).

The monthly safe fish meal consumption limit (CR_mm_), as proposed by the US EPA [37], was applied to estimate the maximum number of safe meals (servings) per month for each food category. The calculation was performed using the following equation:

$${\text{CR}}_{\text{mm}}=\frac{{\text{CR}}_{\text{lim}}\times T}{\text{MS}}.$$

In Taiwan, dietary evaluations typically rely on exchange serving sizes instead of an average meal size (MS). Consequently, this study used the exchange serving values (g/serving) from the Ministry of Health and Welfare [38] as substitutes for MS in the CR_mm_ calculations. To align with international assessments, an average fish meal size of 0.065 kg per meal for children, as recommended by the US EPA [37], was also employed to estimate the CR_mm_ related to fish intake. Here, T was set to 30.44 days per month [37], and MS was given either in g/serving (for the Taiwan-based analysis) or as 0.065 kg/meal (for the US EPA-related comparison).

### 2.5 Nutrient contents of foods

As eggs and fish exhibited the highest HBV_Se_ values, we further analyzed the nutrient compositions and risk-benefit assessments associated with consuming eggs, marine fish, and freshwater fish to meet the daily AI_Se_, equivalent to a daily intake of 20 µg of Se. The food nutrient composition data were obtained from the TFDA food nutrient database [39].

### 2.6 Statistical analysis

Continuous variables are presented as the mean ± standard deviation (SD), while categorical variables are expressed as counts and percentages. Internal Hg exposure levels, assessed using hair Hg as a biomarker, were categorized into high- and low-exposure groups according to the US EPA recommended dose of 1 µg/g in hair. A multivariable regression was utilized to examine relationships between the percentage of net AI_Se_ and growth performance. Covariates with a *p* value of <0.2 in the univariate regression analysis were subsequently included in the multivariable model. The final model, with growth outcome indicators as dependent variables and percentages of net AI_Se_ as independent variables, was adjusted for high or low Hg exposure, gender, income, parity, and age at measurement. All analyses were carried out using SAS 9.3 software for Windows (SAS Institute, Cary, NC, USA).

## 3. Results

### 3.1 Population characteristics and Se and Hg concentrations in food

Population characteristics, including demographics, family characteristics, and some anthropometric-related measurements like height, weight, and hair Hg concentrations, are shown in Table 1. Among the 349 subjects, the average age was 2.9 ± 1.8 years. Of these, 54.0% were male and 46.0% were female. Less than half of the families (42.1%) had a monthly income exceeding US$3300. The average height of the children was 91.7 ± 17.1 cm, while their average weight was 13.9 ± 6.3 kg. The mean Hg concentration in the children’s hair was 1.11 ± 1.15 µg/g, with 42.4% of the children having hair Hg levels exceeding the US EPA’s recommended reference dose of 1 µg/g [7].

**Table 1:** Demographic characteristics (*N* = 349)

**Characteristic**	**Mean ± SD or *n* (%)**
Gender	
Male	188 (54.0%)
Female	161 (46.0%)
Age of the children (years)	2.9 ± 1.8
Parity of the mother	
1	211 (60.5%)
2	120 (34.5%)
≥3	18 (5.0%)
Family income (US$/month)	
<2300	101 (28.9%)
2300∼3300	101 (28.9%)
>3300	147 (42.1%)
Height of children (cm)	91.7 ± 17.1
Weight of children (kg)	13.9 ± 6.3
Hair Hg level of children (µg/g)	1.11 ± 1.15
≤1 µg/g	201 (57.6%)
>1 µg/g	148 (42.4%)

Abbreviations: SD, standard deviation; Hg, mercury.

As to results of Se and Hg concentrations, the Se:Hg molar ratio and HBV_Se_ in each food item are presented in Table 2. Se concentrations were highest in eggs (0.521 ± 0.045 mg/kg wet weight (ww)) and fish (0.408 ± 0.192 mg/kg ww), whereas lowest values were found in fruits (0.002 ± 0.002 mg/kg ww). Se concentrations were higher in marine fish (0.452 ± 0.199 mg/kg ww) compared to freshwater fish (0.309 ± 0.149 mg/kg ww). Hg concentrations in foods were in descending order of fish > shellfish > grains and related products > eggs. Specifically, the mean Hg concentration in fish was 0.078 ± 0.072 mg/kg ww. Within fish, Hg levels in marine species (mean: 0.093 ± 0.081 mg/kg, ww) were 2.2-times higher than those in freshwater species (mean: 0.044 ± 0.033 mg/kg, ww). Hg contents in eggs were 5.5-times lower than that in fish. The lowest Hg concentrations were observed in non-leafy vegetables, leafy vegetables, and fruits.

**Table 2:** Mercury and selenium concentrations, molar ratios, and health benefit value (HBV_Se_) in different foods

**Category**	***n***	**Se** **(mg/kg)**	**Se** **(µmol/kg)**	**Hg** **(mg/kg)**	**Hg** **(µmol/kg)**	**Ratio Se:Hg**	**HBV_Se_**
Eggs	6	0.521 ± 0.045	6.486 ± 0.570	0.014 ± 0.004	0.069 ± 0.021	94.27	6.48
Fish	13	0.408 ± 0.192	5.170 ± 2.710	0.078 ± 0.072	0.389 ± 0.359	13.29	5.14
Marine fish	9	0.452 ± 0.199	5.726 ± 2.520	0.093 ± 0.081	0.464 ± 0.404	12.35	5.69
Freshwater fish	4	0.309 ± 0.149	3.920 ± 1.887	0.044 ± 0.033	0.219 ± 0.165	17.87	3.91
Fruits	13	0.002 ± 0.002	0.020 ± 0.019	0.005 ± 0.001	0.023 ± 0.006	0.89	−0.01
Grains and related products	10	0.158 ± 0.186	2.004 ± 2.362	0.015 ± 0.010	0.076 ± 0.048	26.54	2.00
Leafy vegetables	9	0.006 ± 0.005	0.075 ± 0.058	0.005 ± 0.005	0.022 ± 0.023	3.37	0.07
Legumes and related products	7	0.086 ± 0.063	1.094 ± 0.798	0.009 ± 0.006	0.043 ± 0.030	25.66	1.09
Meat and meat products	18	0.255 ± 0.099	3.232 ± 1.428	0.011 ± 0.003	0.055 ± 0.019	58.29	3.23
Milk and dairy products	10	0.063 ± 0.055	0.795 ± 0.706	0.007 ± 0.004	0.034 ± 0.022	23.47	0.79
Non-leafy vegetables	13	0.028 ± 0.069	0.353 ± 0.878	0.004 ± 0.003	0.020 ± 0.015	17.59	0.35
Rice^a^	4	0.036 ± 0.039	0.460 ± 0.494	0.025 ± 0.012	0.122 ± 0.060	3.77	0.43
Shellfish	5	0.306 ± 0.113	3.878 ± 1.429	0.022 ± 0.023	0.108 ± 0.114	35.96	3.88
Total	108	0.166 ± 0.189	2.101 ± 2.375	0.018 ± 0.034	0.088 ± 0.167	23.85	2.09

^a^Dry weight. Abbreviation: Se, selenium; Hg, mercury. Marine and freshwater fish were classified according to the commonly consumed species identified in the dietary survey (see Table S1 for details).

### 3.2 Risk-benefit assessment and related AI_Se_ percentages

A positive HBV_Se_ value and an Se:Hg molar ratio of >1 are indicators of a wholesome food item. Maximum HBV_Se_ values were found in eggs and fish, at 6.48 and 5.14, respectively. Negative HBV_Se_ values and Se:Hg molar ratios below 1 were found only in fruits, but their low Se contribution was offset by Se-rich foods such as eggs and fish, leading to an overall positive dietary Se balance. Higher Se:Hg molar ratios were observed in meat, poultry, shellfish, and grains compared to fish. Nonetheless, the notably elevated HBV_Se_ value in fish could still be considered healthy and a good source of Se (Table 2).

**Fig. 1:**
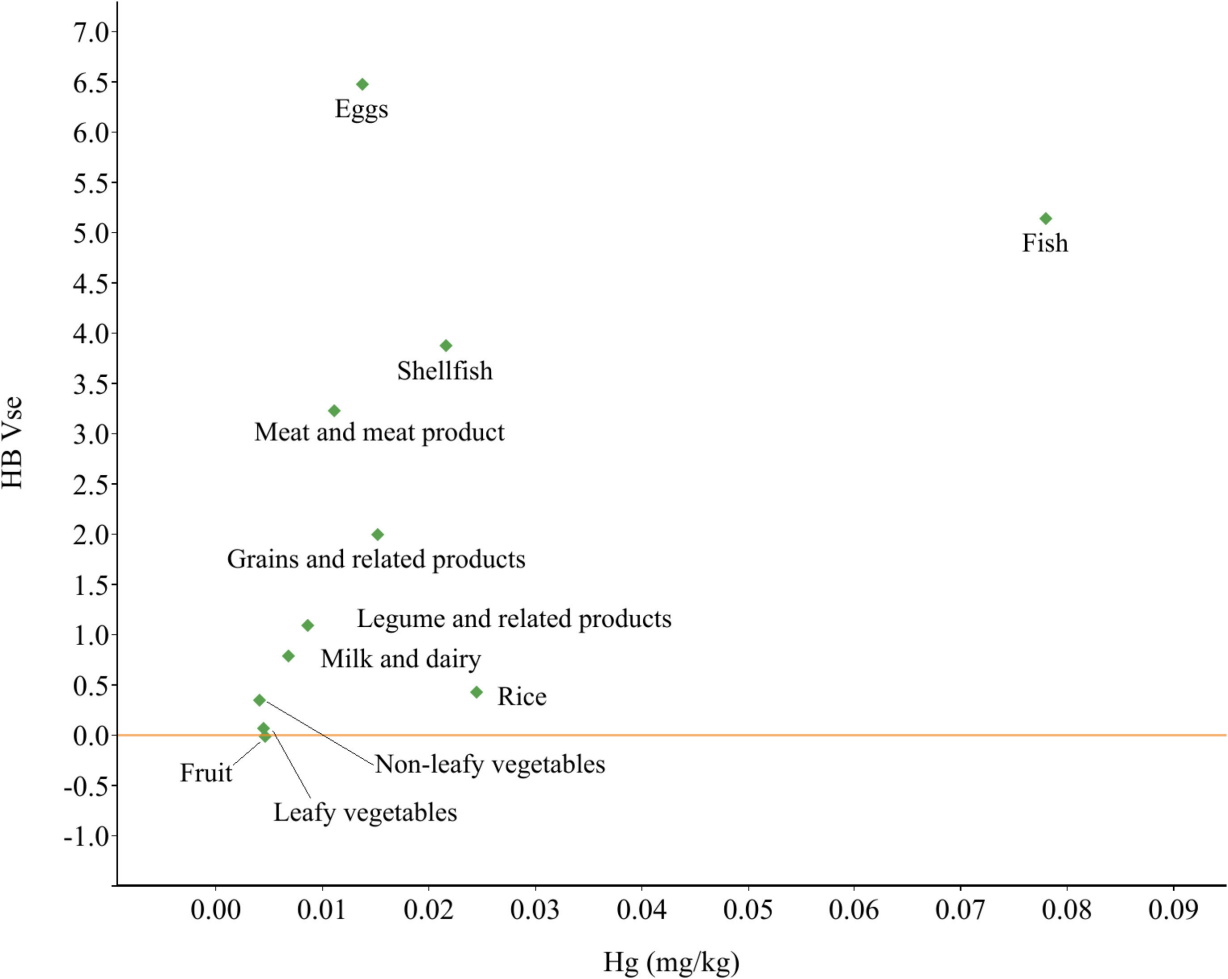
Relationship between mercury (Hg) concentrations (mg/kg) and selenium’s health benefit value (HBV_Se_) in food.

Figure 1 illustrates the association between Hg concentrations (mg/kg) and HBV_Se_ values in each food. The findings showed that eggs are abundant in Se and have lower levels of Hg. This suggests that consuming eggs could increase Se levels despite some loss due to Hg. Even though fish had the highest Hg content, its positive HBV_Se_ value indicated that consuming fish could provide a surplus of Se and help reduce risks of Hg exposure.

Table 3 reveals the dietary Se EDI, the percentage of meeting the AI_Se_, and the corresponding Se EDI and AI_Se_ percentages after considering Hg intake levels of children. Daily egg, milk, and dairy consumption provided approximately 87%–90% of AI_Se_ among preschool children in Taiwan. Even taking into account the potential loss of Se due to Hg exposure, children who consumed these foods can still meet 80%–84% of the AI_Se_. Plant-based foods like fruits, leafy vegetables, and non-leafy vegetables had lower percentages of AI_Se_, ranging 1.2%–9.3%. Fruit consumption fell below recommended levels for Se intake due to their limited Se content, but this does not pose a dietary risk because other food groups supply enough Se to maintain a healthy nutritional balance.

**Table 3:** Estimated selenium intake, adequate intake compliance, and adjustments for mercury exposure in children

**Category**	**EDI_Se_** **(µg/day)**	**EDI_Se_/AI_Se_** **(%)**	**EDI_Se_-EDI_Hg_** **(µg/day)**	**(EDI_Se_ − EDI_Hg_)/AI_Se_** **(%)**
Eggs	17.37	86.86	16.81	84.04
Fish	9.41	47.06	7.52	37.61
Fruits	0.24	1.19	−0.51	−2.53
Grains and related products	15.79	78.96	14.27	71.33
Leafy vegetables	0.71	3.55	0.11	0.54
Legumes and related products	3.26	16.29	2.88	14.40
Meat and meat products	12.63	63.17	12.04	60.20
Milk and dairy products	17.99	89.98	15.99	79.94
Non-leafy vegetables	1.86	9.32	1.59	7.98
Rice	1.37	6.86	1.28	6.40
Shellfish	3.24	16.20	3.01	15.03

The adequate intake of selenium (AI_Se_) for preschool children is 20 µg/day (EFSA, 2014; HPA, 2022). Abbreviation: EDI, estimated daily intake; Se, selenium; Hg, mercury.

Table S2 provides the estimated maximum safe daily intake (CR_lim_) and monthly safe consumption limits (CR_mm_) for Hg and Se across various food categories. When expressed per serving (based on Taiwan’s MOHW exchange portion sizes), fish had the smallest monthly safe consumption limits among all food categories. The estimated CR_mm_ values were approximately 13 servings per month for marine fish and 27 servings per month for freshwater fish. In contrast, Se-based CR_mm_ values were significantly higher, exceeding 100 servings per month for fish. However, eggs showed the lowest Se-based CR_mm_ at 67.5 servings per month. The US EPA [37] indicates that fish consumption is generally of no health concern if it exceeds 16 meals monthly. In this research, the calculated CR_mm_ values (based on the US EPA meal size of 0.065 kg per meal) were around seven for marine fish and 14.8 for freshwater fish. Both values are below the restricted limit, suggesting that consumption of these fish, particularly marine types, should be moderated to prevent potential Hg overexposure.

### 3.3 Relationship between dietary Se surplus and growth performance

**Fig. 2:**
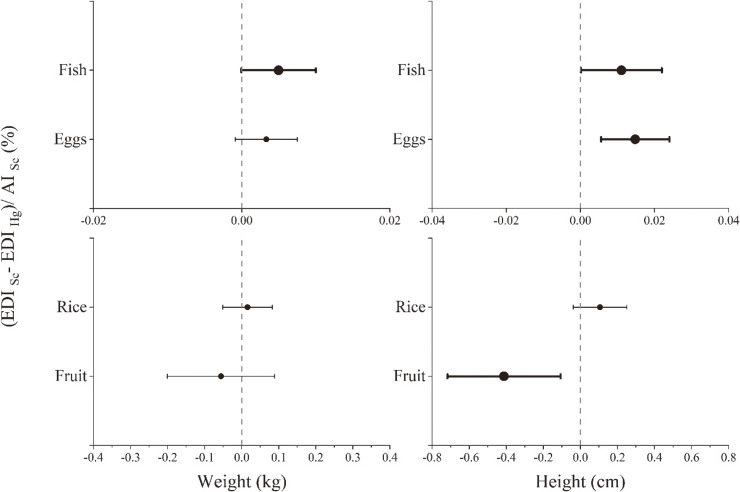
Associations between dietary selenium (Se) adequacy and growth outcomes in young children. Abbreviations: EDI, estimated daily intake; Hg, mercury; AI, adequate intake. The model was adjusted for high or low Hg exposure (using hair Hg as a biomarker categorized into high- and low-exposure groups according to the US EPA recommended dose of 1 µg/g in hair), gender, income, parity, and age at measurement.

A multivariable analysis adjusted for high or low Hg exposure, gender, income, parity, and age at measurement was employed to examine the correlation between the percentage of dietary Se surplus meeting the AI (i.e. dietary AI_Se_ after subtracting Se intake from Hg intake) and growth performance of younger children (Fig. 2). Height performance was positively correlated with the percentage of Se meeting the daily AI through consumption of fish (β = 0.01, 95% confidence interval (CI): −0.00001 to 0.02) and eggs (β = 0.01, 95% CI: 0.01 to 0.02). The dietary Se status from consuming fish was marginally associated with increases in weight (Fig. 2) and the WAZ (Supplemental Fig. 2). Despite high Hg levels (hair Hg >1 µg/g), children exhibited better height and weight development, likely due to the protective effects of Se against Hg exposure. The percentage of dietary Se surplus meeting the AI from fruit was negative, with an average value of −2.53%, and was negatively associated with height (β = −0.41, 95% CI: −0.72 to −0.11). Daily fruit consumption alone contributes little Se, which could decrease height performance. No significant association correlations were estimated for dietary Se intake from rice with weight or height.

### 3.4 Nutrient contents from fish and eggs

**Fig. 3:**
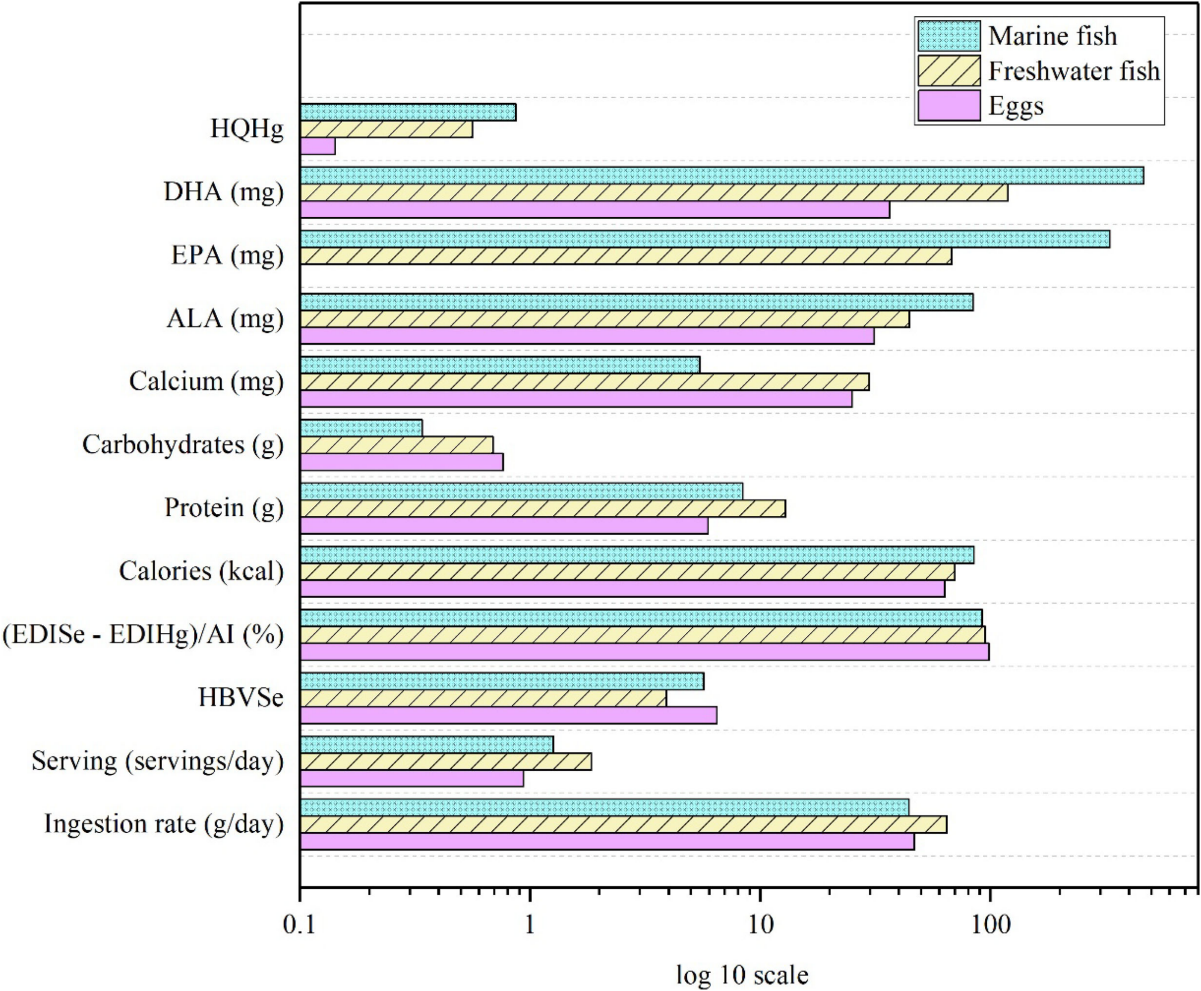
Health benefits, risks, and nutritional contributions of eggs and fish to selenium (Se) intake. Abbreviations: HQ, hazard quotient; Hg, mercury; DHA, docosahexaenoic acid; EPA, eicosapentaenoic acid; ALA, α-linolenic acid; EDI, estimated daily intake; AI, adequate intake; HBV_Se_, selenium’s health benefit value.

Our findings highlighted the impacts of Se consumption from fish and eggs on children’s height and weight. We compared the intake of three food types—marine fish, freshwater fish, and eggs—on meeting the recommended daily Se intake for preschoolers, evaluating serving sizes, nutritional contents, and risks of Hg exposure (Fig. 3). Preschool children can meet their daily Se intake by consuming either one egg, 1.8 servings of freshwater fish, or 1.3 servings of marine fish per day, according to our analysis. Proteins, carbohydrates, calories, and calcium are important nutrients for growth performance [40]. Due to larger serving sizes, protein intake from freshwater fish was twice as high as that from eggs, while carbohydrate intake from freshwater fish was comparable to that from eggs. Caloric intake levels from all three food types were similar. Marine fish provide 4.5–5.4-times less calcium than the other two food types but contain significantly higher levels of ω-3 PUFAs, including docosahexaenoic acid (DHA; 22:6 ω-3), eicosapentaenoic acid (EPA; 20:5 ω-3), and α-linolenic acid (ALA; 18:3 ω-3), at levels 2.6–12-times higher than those found in freshwater fish and eggs. ω-3 PUFAs are critical nutrients associated with a potentially reduced risk of preterm birth and childhood allergic diseases, greater postnatal growth, and beneficial effects on neurodevelopment [41].

Preschool children consuming any of the three foods listed that meet the recommended daily Se intake did not face risks. However, due to elevated Hg levels, particularly in marine fish, the risk linked to fish consumption was notably higher than that of eggs. Nonetheless, our risk-benefit assessment indicated that fish intake remains beneficial for preschoolers’ health.

## 4. Discussion

According to our findings, 42.4% of Taiwanese children in our study had hair Hg concentrations exceeding the US EPA’s reference dose of 1 µg/g, indicating that Hg exposure among preschool children in Taiwan remains a significant concern. The risk-benefit assessment revealed that eggs and fish are superior sources of Se and are better than other animal-based foods. Dietary Se from consuming fish and eggs positively influenced weight and height development. Although fruits have relatively low HBV_Se_ values, an unbalanced diet with excessive fruit intake could slightly reduce Se intake and indirectly affect growth performance. This effect is likely negligible when Se-rich foods are regularly consumed.

### 4.1 Se and Hg concentrations in foods

Based on our studies and consistent with previous research by Moatkhef et al. [42], we found that eggs and fish contained the highest concentrations of Se, whereas fruits and vegetables had the lowest levels. This can be attributed to the positive correlation between the Se content and protein in these foods [24, 42]. Additionally, most fruits have a water content of over 80%, which results in highly diluted Se levels in fruit samples [43]. Therefore, the majority of dietary Se is sourced from major protein-rich foods, and its bioavailability is primarily determined by the presence of Se-containing proteins in those foods [24].

Our analysis showed that the mean Se concentration in fish was 0.408 ± 0.192 mg/kg ww. This result was similar to those obtained in demersal fish from the eastern part of the Adriatic Sea, with a mean value of 0.49 mg/kg ww [44] and in southern India, which ranged 0.203–0.470 mg/kg ww [25]. On the other hand, it was lower compared to levels analyzed in Taiwan in our previous survey, with a mean value of 1.4 mg/kg ww [45], and in coastal areas of the East China Sea, with a mean value of 0.62 mg/kg ww [31]. Variations in Se contents in fish are due to differences in species, habitats, individual sizes, and feeding habits [25], leading to these observed differences. Our findings showed that Se concentrations were higher in marine fish compared to freshwater fish.

The mean Se level in eggs was 0.521 ± 0.045 mg/kg ww, which was comparable to values from total diet studies (TDSs) conducted in the US [46] and in China [47]. This finding was much higher than those reported in Korea, with a mean value of 0.267 mg/kg ww [48] and in China, with a mean value of 0.13 mg/kg ww [49]. However, Moatkhef et al. [42] revealed that the Se value in eggs in Egypt was 1.42 µg/g. The elevated level of Se in eggs in Egypt can be attributed to Se-enriched animal feed practices. Our findings revealed that Se contents in plant-based food such as vegetables, fruits, rice, and grains ranged 0.002–0.158 mg/kg and were consistent with previous results [46, 47, 49].

Among all of the food samples analyzed, the highest Hg concentrations were found in fish, particularly in marine species. However, Hg levels observed in the fish in our study did not exceed the Codex guideline threshold for MeHg of 0.5 mg/kg. In our analysis, the mean Hg concentration in fish was 0.078 ± 0.072 mg/kg ww. Zou et al. [31] analyzed Hg concentrations in wild commercial fish in coastal areas of the East China Sea and found an average value of 0.068 mg/kg, which was comparable to our findings. Arisekar et al. [25] reported Hg concentrations in muscle tissues of marine fish from the southeast coast of India which ranged 0.024–0.106 mg/kg. They indicated that tuna exhibited the highest Hg levels because of its pelagic habitat and carnivorous trophic habit. Our analysis revealed that Hg concentrations in fish varied according to their feeding behavior, with carnivorous fish showing the highest levels (0.09 ± 0.04 mg/kg ww), followed by omnivorous fish (0.07 ± 0.07 mg/kg ww) and planktivorous filter-feeding fish (0.02 ± 0.04 mg/kg ww). Furthermore, we found that Hg levels in marine fish were 2.2-times higher than those in freshwater fish. Among the examined samples, 54% exhibited Hg concentrations higher than 0.05 mg/kg ww. In comparison, Hsi et al. [50] observed that 70% of samples from fish markets in Taiwan had MeHg concentrations exceeding 0.05 mg/kg ww, a value 1.3-times greater than that reported in our study. This may indicate a declining trend in Hg levels in fish in Taiwan. Hg concentrations in vegetables and fruits, ranging 0.004–0.005 mg/kg ww, were consistent with those reported in previous surveys [46, 47, 49].

### 4.2 Hg concentrations in hair

The mean hair Hg concentration among preschool children in our study was 1.11 ± 1.15 µg/g, which was comparable to those reported in Spanish [12] and Japanese children [51], but notably higher than levels measured in European children [52] and US children from the NHANES 1999–2000 cohort [17]. Fish consumption is a well-established major source of internal Hg exposure, accounting for approximately 80%–90% of total intake [53]. Given Taiwan’s island geography and high frequency of seafood consumption, this may explain why the Hg body burden in Taiwanese children was similar to that of populations in other high-fish-consumption countries such as Japan and Spain [54]. Importantly, 42.4% of the children surpassed the US EPA reference level of 1 µg/g, highlighting the need for ongoing monitoring of Hg exposure in Taiwanese children and emphasizing the importance of risk–benefit evaluation of dietary Hg intake.

### 4.3 HBV_Se_ in foods

Values of the HBV_Se_ in foods varied from −0.01 to 6.48, with minimum values found in fruits and maximum values in eggs. All foods except for fruit had HBV_Se_ values of >0, indicating that consumption of these foods was a good net source of surplus Se [26]. Our analysis of the HBV_Se_ in eggs was comparable to TDS results conducted in China [47] and the US [46]. According to Zhang et al. [49], the HBV_Se_ in eggs was 1.6, which was lower than our findings. This discrepancy is attributed to the lower Se content in eggs analyzed by Zhang et al. The HBV_Se_ for fish was 5.14. Comparable studies by Arisekar et al. [25] reported values of 2.48–5.95, and Grgec et al. [44] found values of 3.28–9.26. HBV_Se_ levels in fish vary depending on the species, habitats, and feeding habits. Zou et al. [31] revealed that high-trophic fish had significantly higher HBV_Se_ levels compared to low-trophic and mid-trophic fish. Furthermore, benthic fish exhibited significantly higher HBV_Se_ levels than pelagic fish. Our results indicated that marine fish presented higher HBV_Se_ levels than freshwater fish. Due to the well-documented antagonistic effects between Se and Hg, the HBV_Se_ index is a reliable indicator for distinguishing impacts of Hg exposure from those of dietary Se intake [26, 55]. This index more accurately reflects effective net Se contents in commonly consumed foods. Ralston and Raymond [27] advocated for implementation of higher dietary Hg exposures with positive HBV_Se_ levels for consumers, which would improve maternal and fetal Se statuses while providing nutritional benefits for neurological outcomes. Our findings suggest that the ingestion of Se in marine fish may provide protection against the toxic effects of Hg. The HBV_Se_ value for fruits was below 1, indicating a relatively low Se contribution. However, this does not imply a negative health impact, as the cumulative HBV_Se_ across all food groups remained positive. Moderate fruit consumption within a balanced diet should not compromise the Se status or its protective effects against Hg exposure.

### 4.4 Recommended Se and Hg intake levels from fish and egg ingestion

A comparison of CR_mm_ values for Hg and Se highlights fish as the primary dietary contributor to Hg exposure among preschool children in Taiwan. Estimated CR_mm_ values of seven meals per month for marine fish and 14.8 meals per month for freshwater fish were both below the US EPA’s recommended safety threshold of 16 meals per month [37], suggesting that frequent consumption, particularly of marine species, may increase the risk of Hg overexposure in preschoolers. In contrast, Se-based CR_mm_ values exceeded 100 servings per month for most food categories, indicating a wide margin of safety for Se intake. The relatively lower Se-based CR_mm_ observed for eggs (67.5 servings per month) suggests that consuming up to two eggs daily (roughly two servings) remains within the safe range and does not pose a risk of Se overexposure.

Further comparisons of marine fish, freshwater fish, and eggs showed that eggs required smaller serving sizes to meet the recommended Se intake for preschoolers due to their higher Se content. Although fish consumption generally results in greater Hg exposure than eggs, marine fish provide substantially higher amounts of protein and ω-3 PUFAs, which are essential for growth and neurodevelopment.

Hg exhibits a strong binding affinity for Se, forming Hg-Se complexes that can reduce the bioavailability of Se for selenoprotein synthesis. However, relatively high Se contents in marine fish may help maintain an adequate Se status and sustain selenoenzyme activity, provided that Hg exposure remains within safe limits. Previous studies showed that some predatory species, like tuna, swordfish, and blue sharks, tend to accumulate higher levels of Hg. Therefore, their consumption should be limited, particularly among vulnerable populations such as pregnant women and children [4, 56–58].

In our study population, preschool children were not frequent consumers of those predatory fish species. Given this dietary pattern, marine fish can serve as a beneficial dietary component for preschool children when consumed in moderation, balancing their nutritional advantages against potential Hg-related risks. These findings underscore the importance of balancing nutritional benefits with potential contaminant risks when establishing dietary recommendations for children. Continuous monitoring of Hg concentrations in locally consumed fish and clear public education on safe consumption frequencies are essential to ensure that children continue to obtain nutritional benefits while minimizing Hg exposure.

### 4.5 Se intake for growth performance

Our research demonstrated that dietary Se obtained from consuming fish and eggs positively influenced weight and height development in preschoolers. Although fruits contribute minimally to overall Se intake, consuming Se-rich foods like eggs and fish compensates for this and promotes healthy growth. Se acts as a growth factor, supports antioxidant systems, and aids thyroid function [23]. A Se deficiency has detrimental roles in growth and development processes of children [59], such as increasing susceptibility to infections [60], raising the incidence of preterm births [61], elevating the risk of allergic diseases [62], and fostering suboptimal neurodevelopment [36]. Moody et al. [63] found that lower height in healthy children was associated with higher blood lead and lower blood Se concentrations. Despite having high Hg levels (hair Hg >1 µg/g), children in this study demonstrated adequate height and weight development, likely due to Se’s protective effects against Hg exposure. Therefore, ensuring adequate Se intake through a balanced diet is essential for promoting healthy growth and development in preschool children.

A limitation of this study is that the Hg analysis was only performed for THg rather than speciated forms, such as MeHg and inorganic mercury (IHg). The proportion of MeHg can considerably vary among different food types, particularly among fish, shellfish, and plant-based foods. Therefore, the estimated exposure levels presented in this study might not fully reflect the variability in bioavailable and toxicologically active mercury species. Future studies, including mercury speciation analyses, are warranted to provide a more-accurate assessment of dietary MeHg exposure in children.

## 5. Conclusions

This study provides a comprehensive evaluation of dietary Hg and Se exposure among Taiwanese preschool children. Although 42.4% of the children had hair Hg concentrations exceeding the US EPA reference dose, the overall dietary assessment indicates no evidence of harmful Hg exposure. The health benefit value (HBV_Se_) analysis showed that all food categories, except fruit, contributed positively to Se intake. The minor negative HBV_Se_ observed for fruit does not pose a health concern, as it is compensated by other Se-rich foods in the overall diet. The additive nature of HBV_Se_ across food groups suggests that the net dietary effect for these children was uniformly positive and nutritionally beneficial.

Marine fish and eggs were identified as key dietary sources of Se, with fish additionally providing essential ω-3 PUFAs that support brain growth and neurodevelopment. While marine fish contain higher Hg concentrations than eggs, their relatively high Se and ω-3 PUFA contents help maintain an overall favorable nutritional profile when consumed in moderation. Overall, these findings indicate that the dietary patterns of Taiwanese preschool children provide beneficial levels of Se and ω-3 fatty acids while posing no adverse effects of Hg exposure. A balanced consumption of fish and eggs should therefore be encouraged to support optimal growth and neurodevelopment, accompanied by continuous monitoring of Hg levels in locally consumed foods to ensure dietary safety.

## Supplementary information

**Additional file 1:** **Table S1** Common fish species identified from the dietary survey. **Table S2** Maximum safe daily intake (CR_lim_) and the monthly safe consumption limit (CR_mm_) for Hg and Se across each food category. **Supplemental Fig. S1.** Flowchart of participant recruitment and study design. **Supplemental Fig. S2.** Associations between the percentage of dietary selenium (Se) surplus meeting the adequate intake (AI), weight-for-age z-score (WAZ), and height-for-age z-score (HAZ) for younger children.

## Declarations

### Availability of data and material

Data available on request from the authors.

Data described in the manuscript, code book, and analytic code will be made available upon request pending application.
