# Supplementary material for: A risk-benefit assessment of dietary selenium and its implications in preschool children’s growth performance in Taiwan
Source: Environ Health Prev Med. 2026 Jan 9;31:2. doi: 10.1265/ehpm.25-00128 (PMC12807879; doi:10.1265/ehpm.25-00128)
Supplement: Supplementary file 1 — Additional file 1: Table S1 Common fish species identified from the dietary survey. Table S2 Maximum safe daily intake (CRlim) and the monthly safe consumption limit (CRmm) for Hg and Se across each food category. Supplemental Fig. S1. Flowchart of participant recruitment and study design. Supplemental Fig. S2. Associations between the percentage of dietary selenium (Se) surplus meeting the adequate intake (AI), weight-for-age z-score (WAZ), and height-for-age z-score (HAZ) for younger children. [file ehpm-31-002-s001.docx]

**Table S1** Common fish species identified from the dietary survey

| Category | Representative species (English name) |
| --- | --- |
| Marine species | *Epinephelus* spp. (grouper), *Lutjanus erythropterus* (crimson snapper), *Nemipterus japonicus* (Japanese butterfish bream), *Engraulis japonicus* (whitebait/anchovy fry), *Mugil cephalus* (mullet), *Lates calcarifer* (Asian seabass), *Salmo salar* (salmon), *Gadus morhua* (cod), tuna, *Scomber japonicus* (mackerel), and *Cololabis saira* (Pacific saury) |
| Freshwater species | *Oreochromis* spp. (tilapia) |

**Table S2** Maximum safe daily intake (CR_lim_) and the monthly safe consumption limit (CR_mm_) for Hg and Se across each food category

| Category | Hg (mg/kg) | Se  (mg/kg) | CR_lim_ of Hg (g/day) | CR_lim_ of Se (g/day) | MS  (g/serving)^a^ | MS  (g/meal)^b^ | CR_mm_ of Hg (servings /month) | CR_mm_ of Se  (servings /month) | CR_mm_ of Hg (meals /month) | CR_mm_ of Se (meals /month) |
| --- | --- | --- | --- | --- | --- | --- | --- | --- | --- | --- |
| Eggs | 0.014 | 0.521 | 99.07 | 133.11 | 60 | NA | 50.3 | 67.5 | NA | NA |
| Fish | 0.078 | 0.408 | 17.78 | 169.98 | 35 | 65 | 15.5 | 147.8 | 8.3 | 79.6 |
| Marine fish | 0.093 | 0.452 | 14.91 | 153.43 | 35 | 65 | 13.0 | 133.4 | 7.0 | 71.9 |
| Freshwater fish | 0.044 | 0.309 | 31.52 | 224.43 | 35 | 65 | 27.4 | 195.2 | 14.8 | 105.1 |
| Fruits | 0.005 | 0.002 | 277.40 | 34,675.00 | 100 | NA | 84.4 | 10,555.1 | NA | NA |
| Grains and related products | 0.015 | 0.158 | 92.47 | 438.92 | 80 | NA | 35.2 | 167.0 | NA | NA |
| Leafy vegetables | 0.005 | 0.006 | 277.40 | 11,558.33 | 100 | NA | 84.4 | 3518.4 | NA | NA |
| Legumes and related products | 0.009 | 0.086 | 154.11 | 806.40 | 20 | NA | 234.6 | 1227.3 | NA | NA |
| Meat and meat products | 0.011 | 0.255 | 126.09 | 271.96 | 35 | NA | 109.7 | 236.5 | NA | NA |
| Milk and dairy products | 0.007 | 0.063 | 198.14 | 1100.79 | 240 | NA | 25.1 | 139.6 | NA | NA |
| Non-leafy vegetables | 0.004 | 0.028 | 346.75 | 2476.79 | 100 | NA | 105.6 | 753.9 | NA | NA |
| Rice | 0.025 | 0.036 | 55.48 | 1926.39 | 40 | NA | 42.2 | 1466.0 | NA | NA |
| Shellfish | 0.022 | 0.306 | 63.05 | 226.63 | 65 | NA | 29.5 | 106.1 | NA | NA |

Abbreviation: Se, selenium; Hg, mercury; MS, meal size; NA, not applicable.

**^a^** MS values are expressed in g/serving based on exchange serving sizes recommended by the Ministry of Health and Welfare, Taiwan (MOHW, 2018).

^b^ For fish consumption, MS values are expressed in g/meal (0.065 kg/meal) following United States Environmental Protection Agency (US EPA, 2000) recommendations.

**Figure captions**

Supplemental Fig. S1. Flowchart of participant recruitment and study design.

Supplemental Fig. S2. Associations between the percentage of dietary selenium (Se) surplus meeting the adequate intake (AI), weight-for-age z-score (WAZ), and height-for-age z-score (HAZ) for younger children.


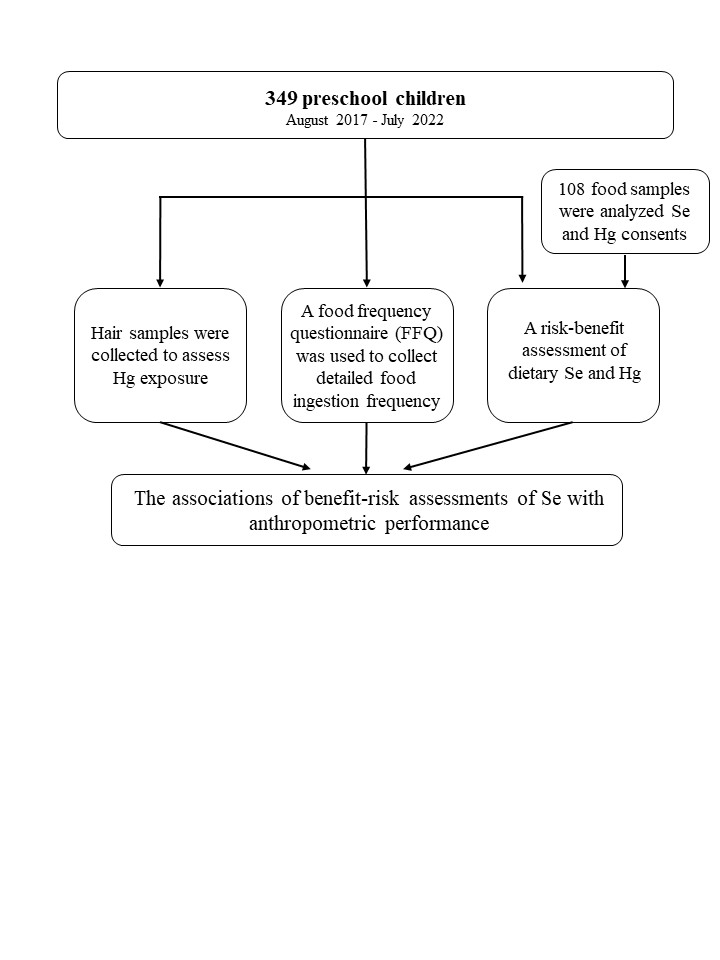


Supplemental Fig. S1. Flowchart of participant recruitment and study design.


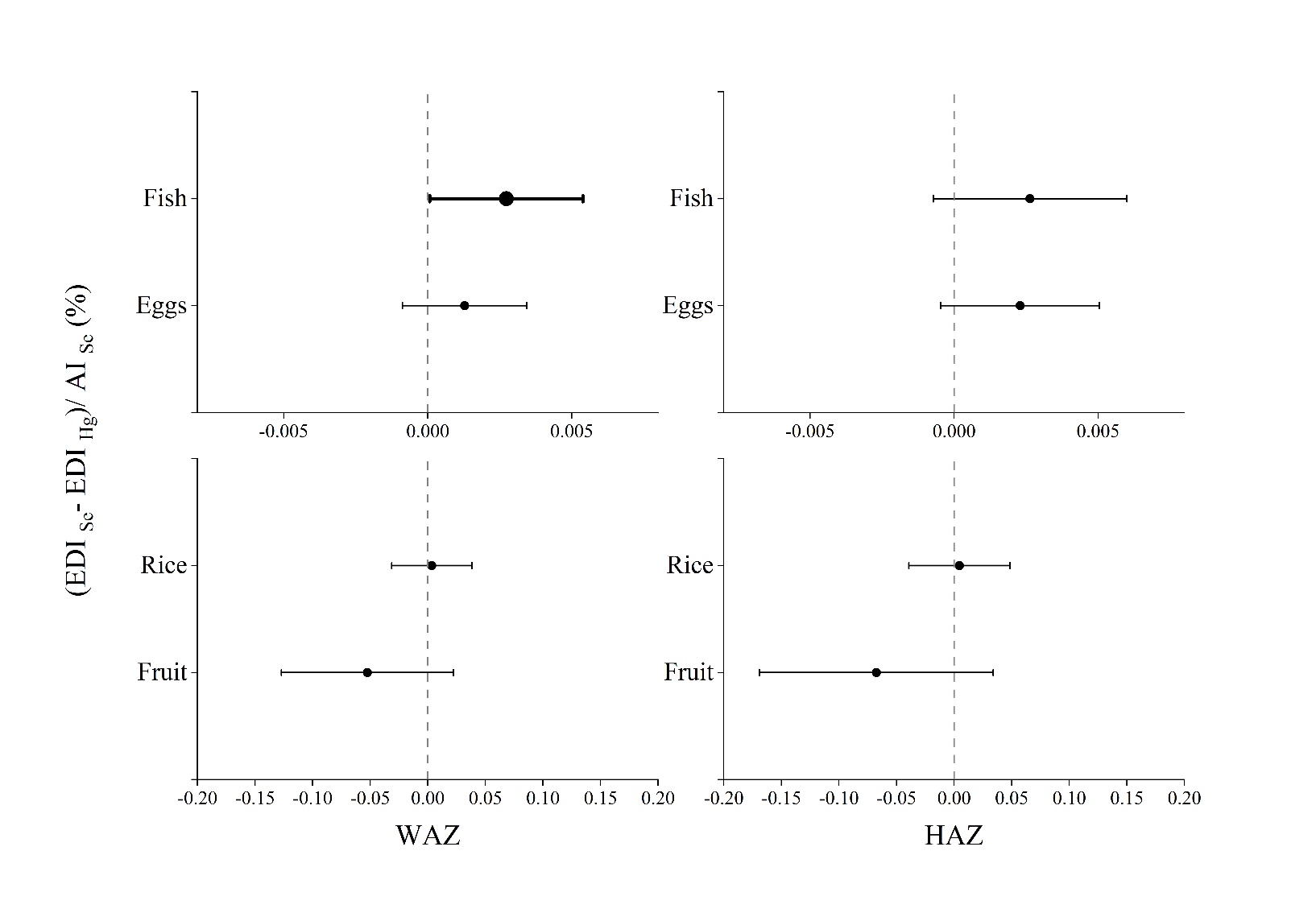


Supplemental Fig. S2. Associations between the percentage of dietary selenium (Se) surplus meeting the adequate intake (AI), weight-for-age z-score (WAZ), and height-for-age z-score (HAZ) for younger children.

The model was adjusted for high or low Hg exposure (using hair Hg as a biomarker categorized into high- and low-exposure groups according to the US EPA recommended dose of 1 μg/g in hair), gender, income, and parity.
